# Activating Receptor Signals Drive Receptor Diversity in Developing Natural Killer Cells

**DOI:** 10.1371/journal.pbio.1002526

**Published:** 2016-08-08

**Authors:** Jacquelyn Freund, Rebecca M. May, Enjun Yang, Hongchuan Li, Matthew McCullen, Bin Zhang, Todd Lenvik, Frank Cichocki, Stephen K. Anderson, Taku Kambayashi

**Affiliations:** 1 Department of Pathology and Laboratory Medicine, Perelman School of Medicine at the University of Pennsylvania, Philadelphia, Pennsylvania, United States of America; 2 Basic Science Program, Leidos Biomedical Research Inc., Frederick National Lab, Frederick, Maryland, United States of America; 3 Cancer and Inflammation Program, Center for Cancer Research, National Cancer Institute, Frederick, Maryland, United States of America; 4 Division of Hematology, Oncology, and Transplantation, Department of Medicine, University of Minnesota, Minneapolis, Minnesota, United States of America; National Jewish Medical and Research Center/Howard Hughes Medical Institute, UNITED STATES

## Abstract

It has recently been appreciated that NK cells exhibit many features reminiscent of adaptive immune cells. Considerable heterogeneity exists with respect to the ligand specificity of individual NK cells and as such, a subset of NK cells can respond, expand, and differentiate into memory-like cells in a ligand-specific manner. MHC I-binding inhibitory receptors, including those belonging to the Ly49 and KIR families, are expressed in a variegated manner, which creates ligand-specific diversity within the NK cell pool. However, how NK cells determine which inhibitory receptors to express on their cell surface during a narrow window of development is largely unknown. In this manuscript, we demonstrate that signals from activating receptors are critical for induction of Ly49 and KIR receptors during NK cell development; activating receptor-derived signals increased the probability of the Ly49 bidirectional Pro1 promoter to transcribe in the forward versus the reverse direction, leading to stable expression of Ly49 receptors in mature NK cells. Our data support a model where the balance of activating and inhibitory receptor signaling in NK cells selects for the induction of appropriate inhibitory receptors during development, which NK cells use to create a diverse pool of ligand-specific NK cells.

## Author Summary

Natural killer (NK) cells are important cells of the immune system, because they kill abnormal cells such as those infected with viruses or have become cancerous. These abnormal cells can lose proteins known as MHC molecules, which are recognized by inhibitory receptors on NK cells. Thus, when an NK cell interacts with a cell with decreased MHC, the NK cell is disinhibited and can kill the target cell. Each NK cell carries a unique assortment of these inhibitory receptors. However, how developing NK cells determine which inhibitory receptors to put on the NK cell’s surface during development is unknown. In this study, we show that signals generated through NK cell activating receptors are important for inducing a subset of inhibitory receptors on NK cells during development. We propose that the NK cell has an increased chance of acquiring an inhibitory receptor until a balance between activating and inhibitory receptor signals is achieved. This process ensures that NK cells can properly detect abnormal cells that have lost MHC.

## Abstract 3

During the development of natural killer (NK) cells, activating receptor signaling drives the acquisition of inhibitory receptors during development, leading to the creation of a diverse pool of ligand-specific NK cells.

## Introduction

Natural killer (NK) cells are innate lymphocytes that play an important role in defense against viral infections and tumor clearance. NK cells express a wide variety of inhibitory and activating receptors, whose downstream signals integrate to dictate a functional response. For example, the Ly49 family of receptors on murine NK cells plays a key role in NK cell function. Inhibitory Ly49 receptors (e.g., Ly49A, Ly49G, Ly49C, and Ly49I) recognize major histocompatibility complex class I (MHC I) and allow NK cells to carry out “missing-self” recognition, a process that eliminates cells with abnormally down-regulated MHC I expression due to certain types of infection or neoplastic transformation [1,2]. Also, the activating receptor Ly49H binds to cytomegalovirus (CMV)-encoded m157 protein, aiding in the clearance of CMV-infected cells. Ly49 receptors are acquired in a sequential and variegated manner during development, which yields a diverse repertoire of NK cells with various Ly49 receptor expression patterns. Since each Ly49 receptor recognizes a subset of MHC I alleles, the Ly49 receptor expression pattern on an individual NK cell determines its target cell specificity. Thus, unlike T and B cells that create antigen-specific diversity by genetic recombination, NK cells generate ligand-specific diversity by acquiring an assortment of inhibitory and activating receptors; however, the mechanisms that regulate NK cell receptor acquisition during development are not well understood.

NK cells commence their acquisition of Ly49 receptors during the immature NK (CD3ε^-^CD122^+^NK1.1^+^DX5^-^) bone marrow (BM) stage [3,4]. Ly49 receptor genes are activated in a specific order, and each receptor possesses a developmental timeframe for the initiation of expression, which is maintained for the lifetime of the NK cell. However, once this window of opportunity passes, the NK cell can never express that Ly49 receptor [5]. Ly49 receptor expression patterns are influenced by polymorphisms in the *Ly49* locus and the MHC haplotype expressed in each strain of mouse. Thus, the fraction of NK cells expressing a particular Ly49 receptor is similar within a given mouse strain. For example, ~10% and ~50% of NK cells in C57BL/6 mice express Ly49A and Ly49G2, respectively.

Ly49 receptor gene transcription is controlled by at least two distinct promoters: Pro1, which is active in immature NK cells, and Pro2, which is critical in maintaining expression in mature NK cells [6–9]. Each Ly49 receptor possesses a unique Pro1 promoter that acts as a bidirectional switch. Transcription factors bind to Pro1 on either the positive (forward) or negative (reverse) strand in a probabilistic manner, thus determining forward or reverse transcription from this promoter. Transcription of Pro1 in the forward direction leads to activation of Pro2 [6,7]. Pro2 can be regulated through DNA methylation, and forward transcription of Pro1 is thought to remove a repressor complex, allowing for acetylation of histones and/or demethylation of DNA at the Pro2 promoter [9,10]. This promotes *Ly49* transcription in mature NK cells and the stable expression of the receptor. Pro1-mediated transcription in the reverse direction results in no Pro2 activity and therefore no Ly49 receptor expression. Thus, the proportion of NK cells expressing a given Ly49 receptor is determined by the probability of the specific Pro1 promoter to transcribe in the forward versus reverse direction.

Two important factors that shape the NK cell inhibitory Ly49 receptor profile are the MHC haplotype and the MHC-binding specificities of the inhibitory receptors themselves [11]. However, the mechanism by which inhibitory receptor specificity and MHC haplotype regulate NK cell receptor acquisition is unclear, especially since inhibitory receptors block (through recruitment of phosphatases such as SHP-1 and SHIP) rather than transmit signals to the NK cell. Mice with NK cells lacking SHP-1 or SHIP display increased proportions of Ly49 receptor-expressing NK cells [12–15], suggesting that inhibitory receptor-induced phosphatase activity attenuates Ly49 receptor acquisition. To explain how this might work, we hypothesized that activating receptor signals are the driving force behind inhibitory receptor acquisition. We propose that MHC I influences the acquisition of inhibitory receptors by blocking this activating signal, which impedes expression of additional inhibitory receptors. This notion is difficult to test by deleting specific activating receptors, as NK cells express numerous activating receptors that utilize various signaling modules. Instead, we took advantage of mice lacking the adaptor molecule SH2 domain-containing leukocyte protein-76 (SLP-76). Although initially described as being dispensable for NK cell activation in IL-2-expanded splenocyte cultures [16,17], subsequent studies have shown that SLP-76 is indeed critical for signal transduction downstream of multiple NK cell activating receptors [17–19]. In this study, we report that activating signals downstream of SLP-76 drive the stable expression of a subset of Ly49 receptors by increasing the probability of forward Ly49 transcription from the bidirectional Pro1 promoter. Our data support a model where competing activating and inhibitory receptor signals determine the probability of Ly49 receptor expression, which ultimately shapes an appropriate inhibitory receptor repertoire during NK cell development.

## Results

### SLP-76-Derived Signals Are Required for Optimal Induction of Ly49 Receptor and KIR Expression by Developing NK Cells

**Fig 1:**
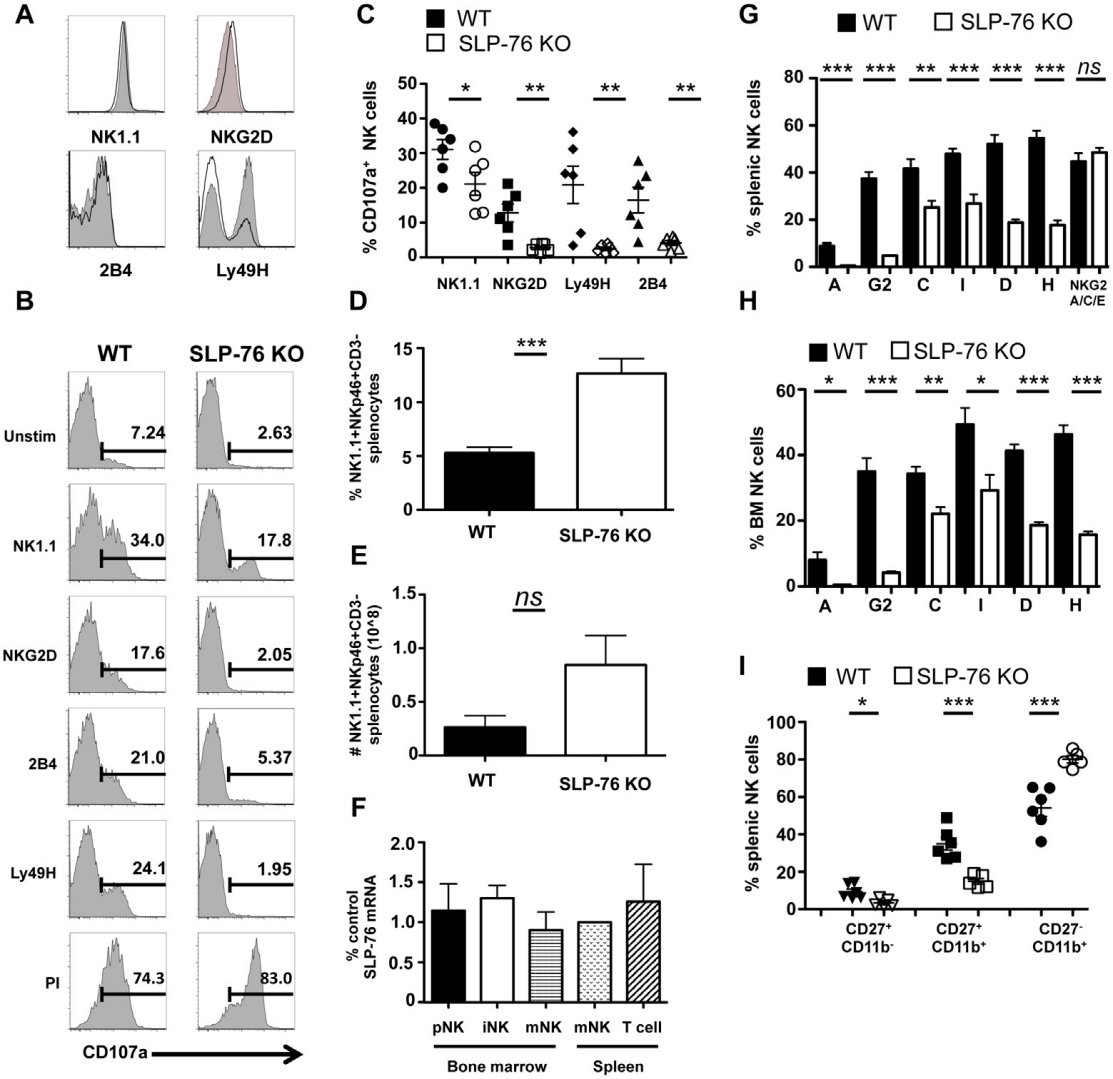
SLP-76 is important for Ly49 receptor expression and activation downstream of multiple NK cell activating receptors. (**A**) Representative histograms of NK cell (gated on NKp46^+^DX5^+^ splenocytes) expression of multiple activating receptors are shown (WT: tint; SLP-76 KO: black line). (**B)** Representative histograms of CD107a expression by WT and SLP-76 KO NK cells post-stimulation from four independent experiments are shown. (**C**) %CD107a expressing NK cells are represented as mean ± standard error of the mean (SEM) of four independent experiments (*n* = 6 mice). Ly49H-stimulated NK cells are gated on Ly49H^+^ cells. (**D**) The percent and (**E**) total number of splenic NK cells (NK1.1^+^NKp46^+^ CD3ε^−^) were calculated and represented as mean percent positive ± SEM of *n* = 4–5 mice/group. (**F**) qPCR was performed for SLP-76 (*lcp2)* and GAPDH (housekeeping gene) with RNA from sorted BM NK cells [CD3ε ^−^CD122^+^NK1.1^−^ (pNK), CD3ε ^−^CD122^+^NK1.1^+^DX5^−^ (iNK), CD3ε ^−^NK1.1^+^DX5^+^ (mNK)], splenic CD3ε ^−^NK1.1^+^DX5^+^ (mNK) and splenic T cells (CD3ε ^+^). Data are from three experimental replicates and is graphed as % of control (splenic mNK). *p < 0.05, **p < 0.01, and ***p < 0.001 by paired student’s *t* test. (**G**) Splenic and (**H)** BM NK cells from WT (black bars) and SLP-76 KO (white bars) mice were assessed for expression of Ly49 inhibitory receptors (Ly49A, Ly49G2, Ly49C, Ly49I), Ly49 activating receptors (Ly49D and Ly49H), and NKG2A/C/E. The proportion of receptor-expressing NK cells from multiple mice is represented as mean percent positive ± SEM of *n* = 4–5 mice/group. (**I)** CD27 and CD11b expression by WT (black) and SLP-76 KO (white) NK cells is represented as percent positive ± SEM of *n* = 5–6 mice. *p < 0.05, **p < 0.01, ***p < 0.001, and ns = not significant by student’s *t* test. See S1 Data for raw data.

To test whether NK cell effector function following activation is dependent on SLP-76, wild-type (WT) and SLP-76 knockout (KO) NK cells were stimulated through three distinct activating receptor families (ITAM-dependent: NK1.1, Ly49H; costimulatory-like: NKG2D; SAP-dependent: 2B4). We found that the baseline expression level (MFI) of all activating receptors on SLP-76 KO NK cells was comparable to controls (Fig 1A). Upon stimulation with antibodies (Abs) against the various activating receptors, SLP-76 KO NK cells were significantly defective in degranulation (as measured by surface CD107a) compared to WT NK cells (Fig 1B and 1C).

Given that SLP-76-mediated signals were critical for NK cell function downstream of multiple activating receptors, we next determined if SLP-76-mediated signals impacted NK cell development. The percentage and absolute number of splenic NK cells in SLP-76 KO mice were higher than WT littermate controls (Fig 1D and 1E). This increase in NK cells was most likely a consequence of the increased availability of homeostatic cytokines due to the lack of competing T cells in SLP-76 KO mice. We also examined expression of SLP-76 in all stages of NK cell development and found that SLP-76 mRNA was highly expressed in both the early and late stages of NK cell development (Fig 1F). Next, WT and SLP-76 KO NK cells were analyzed for expression of activating and inhibitory receptors, including the Ly49 family of receptors. A strikingly significant decrease in Ly49 receptor-expressing splenic and BM NK cells was observed in SLP-76 KO mice. This included both inhibitory (Ly49A, Ly49G2, Ly49C, Ly49I) and activating (Ly49D and Ly49H) family members (Fig 1G and 1H). The earliest acquired receptors, Ly49A and Ly49G2, were most affected by the loss of SLP-76 (~90% reduction) compared to Ly49C and Ly49I (~50% reduction). Not all MHC I-binding inhibitory receptors were reduced, as the proportion of CD94/NKG2A expressing NK cells was unaltered in SLP-76 KO mice (Fig 1G).

Since Ly49 receptor acquisition can also occur at later stages of NK cell development, a maturation defect in SLP-76 KO NK cells could be responsible for the phenotype observed. To test this possibility, we assessed splenic NK cell maturation using the cell surface markers CD27 and CD11b [20] and found that NK cells were more developmentally mature in SLP-76 KO mice (increased proportion of CD27^-^CD11b^+^ NK cells; Fig 1I). Moreover, Ly49 receptor expression by SLP-76 KO NK cells was decreased at every stage of splenic maturation compared to WT controls (S1 Fig). These data show that an NK cell maturation defect was not responsible for the reduction in Ly49 receptor expression.

Killer immunoglobulin-like (KIR) receptors on human NK cells are functional orthologs of Ly49 receptors in mice. To test whether SLP-76-derived signals also contributed to KIR acquisition, we differentiated human NK cells from CD34^+^ umbilical cord blood cells transduced with SLP-76 or scrambled shRNA in vitro for 21 d. SLP-76 shRNA transduction resulted in a decrease in SLP-76 expression, which correlated with a reduced ability to activate KIR gene expression (KIR cocktail of KIR2DL1, KIR2DL2/DL3, KIR3DL1) but not CD56 or NKp46 (S2 Fig). These data along with the Ly49 receptor acquisition defect in SLP-76 KO NK cells suggest that NK cells rely on SLP-76-dependent activation signals for a MHC I-binding inhibitory receptor acquisition.

### The Loss of Ly49 Receptor Expression in SLP-76 KO NK Cells Is MHC I Haplotype-Independent

**Fig 2:**
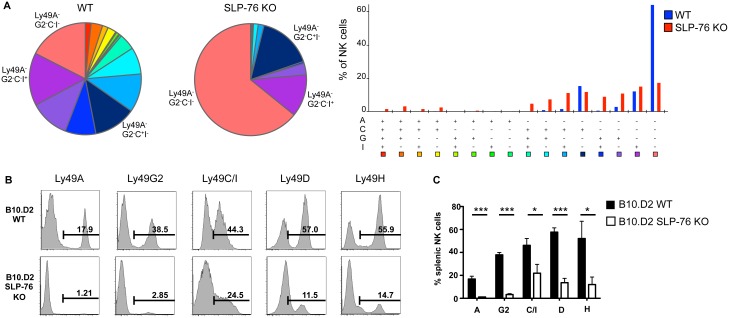
Ly49 receptor expression loss in SLP-76 KO NK cells is independent of MHC I haplotype. **(A)** The coexpression pattern of inhibitory Ly49 receptor in WT vs SLP-76 KO splenic NK cells was assessed through SPICE analysis. (**B)** Representative histograms of Ly49 receptor expression by WT B10.D2 and B10D2.SLP-76 KO splenic NK cells are shown. (**C)** The proportion of Ly49 receptor-expressing NK cells from multiple WT B10.D2 (black bars) and B10D2.SLP-76 KO (white bars) mice is represented as mean percent positive ± SEM of *n* = 3 mice/group. *p < 0.05, ***p < 0.001 by student’s *t* test. See S1 Data for raw data.

To obtain a more global picture of the Ly49 receptor repertoire of SLP-76 KO NK cells, we examined the coexpression pattern of inhibitory Ly49 receptors. This analysis revealed that SLP-76 KO mice have an expanded population of Ly49 receptor-negative NK cells. Although the proportion of NK cells that express Ly49C or Ly49I was reduced in SLP-76 KO mice (Fig 1G), there was relative preservation of Ly49C and Ly49I single-positive NK cells that did not coexpress other Ly49 inhibitory receptors (Fig 2A). Ly49C and Ly49I bind to MHC I (H2-K^b^) in C57BL/6 mice, as opposed to Ly49A and Ly49G2 that bind H-2D^d^ and do not possess ligands in C57BL/6 mice. Since MHC I interactions with Ly49 receptors shape the Ly49 repertoire [12,13,21,22], we wondered whether the relative preservation of Ly49C and Ly49I expression could be related to their ability to bind MHC I in C57BL/6 mice. To test this, we bred SLP-76 KO mice to the B10.D2 mouse strain. If ligand binding were responsible for the preservation of Ly49 receptor expression, the proportion of Ly49A^+^ and Ly49G2^+^ NK cells would be relatively preserved in SLP-76 KO.B10.D2 mice, as B10.D2 mice express H-2D^d^. However, we found that SLP-76 KO-B10.D2 NK cells also displayed a similarly defective Ly49 receptor repertoire as compared to SLP-76 KO NK cells on a H-2^b^ background (Fig 2B and 2C). These data suggest that the Ly49 receptor repertoire defect in SLP-76 KO NK cells is independent of MHC I haplotype.

### SLP-76-Derived Signals Regulate Some, but Not All, Ly49 Receptors in an NK Cell-Intrinsic Manner

**Fig 3:**
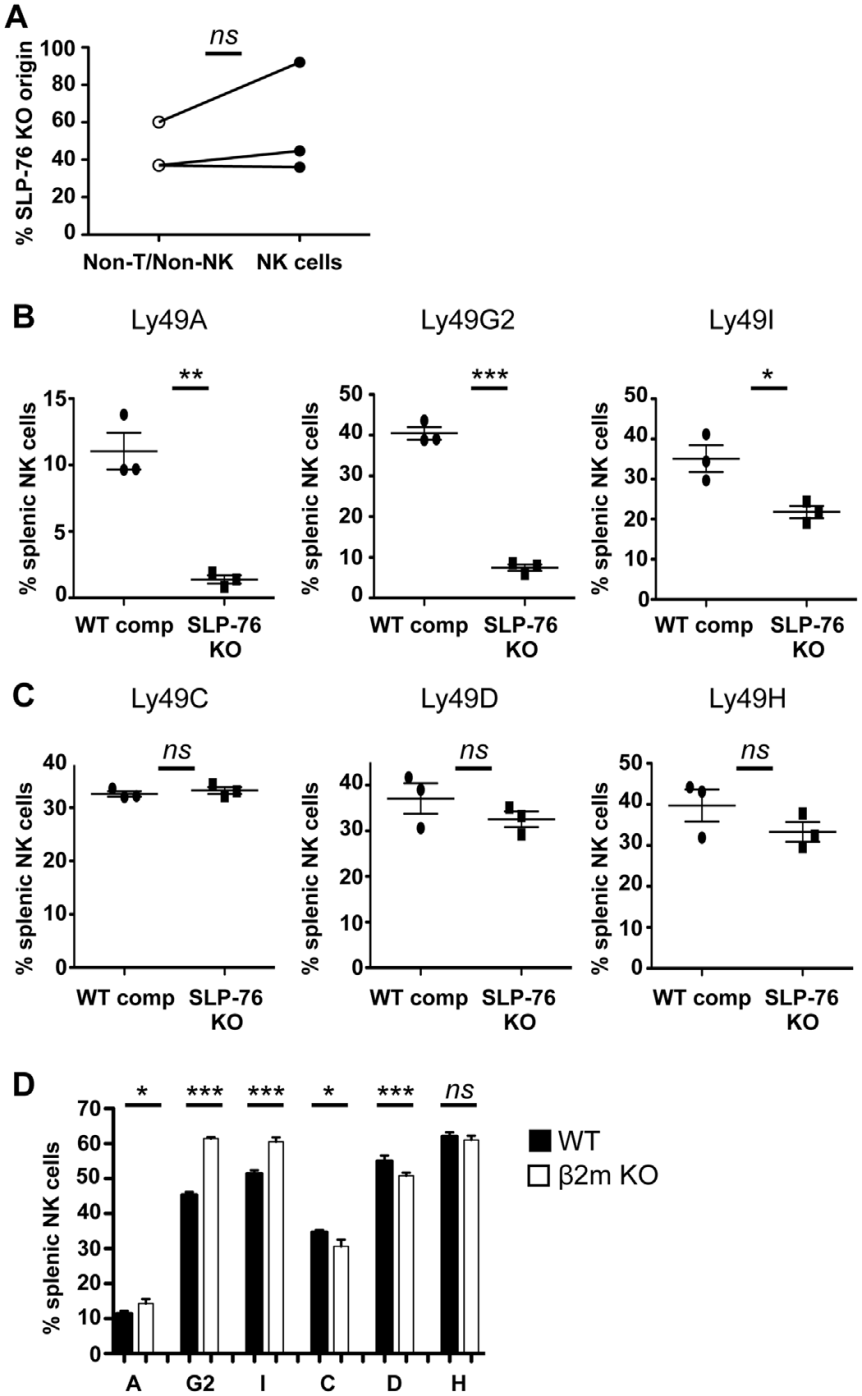
A subset of Ly49 receptors is regulated in an NK cell-intrinsic SLP-76-dependent manner. **(A)** The percentage of Non-T/NK cells and NK cells from WT competitor (CD45.2) was assessed in each mixed BM chimeric mouse. One representative experiment of four experiments is plotted (*n* = 3 mice). ns = not significant by paired student’s *t* test. **(B)** The proportion of Ly49 receptor-expressing NK cells derived from WT or SLP-76 KO BM from mixed BM chimeras was assessed. One representative experiment of four experiments is shown as mean percent positive ± SEM of *n* = 3 mice. *p < 0.05, **p < 0.01, ***p < 0.001, ns = not significant by paired student’s *t* test. **(C)** Ly49 receptor expression on splenic NK cells from WT (black bars) and β2m KO (open bars) mice was assessed and represented as mean percent positive ± SEM of *n* = 10 mice. *p < 0.05, **p < 0.01, ***p < 0.001, and ns = not significant by student’s *t* test. See S1 Data for raw data.

As SLP-76 is expressed in almost all hematopoietic cells, SLP-76 KO mice harbor defects in multiple hematopoietic lineages [23]. Although we predicted that SLP-76-derived signals controlled Ly49 receptor acquisition in an NK cell-intrinsic manner, it was still possible that the defects arose secondary to cell-extrinsic effects. To address this, we generated mixed BM chimeric mice using BM from congenically disparate WT and SLP-76 KO mice mixed at a 2:1 ratio. Ten to twelve weeks after reconstitution, although some variability was seen, the contribution of SLP-76 KO BM to non-T cell/non-NK cells compared to NK cells was similar, suggesting that there was no significant advantage or disadvantage of SLP-76 deficiency in NK cell development (Fig 3A). Consistent with our hypothesis, we found that the proportion of NK cells expressing Ly49A, Ly49G2, and Ly49I was decreased in SLP-76 KO BM compared to WT BM-derived NK cells (Fig 3B). However, no differences in the proportion of Ly49C, Ly49D, and Ly49H expressing NK cells was observed between SLP-76 KO BM and WT BM-derived NK cells (Fig 3B). Thus, although a subset of Ly49 receptors (Ly49A, G2, and I) was regulated in an NK cell-intrinsic manner, Ly49C, Ly49D, and Ly49H were controlled by a SLP-76-dependent, NK cell-extrinsic mechanism.

It has been published that mice deficient in MHC I or inhibitory receptor signaling harbor increased proportions of Ly49-expressing NK cells [12–15]. We hypothesized that the proportion of Ly49 receptor-expressing NK cells is increased in such mice because activation signals are unopposed by MHC-binding inhibitory receptors during NK cell development. The NK cell-intrinsic regulation of some, but not all, Ly49 receptors provided us with an opportunity to test this hypothesis, as we would predict that only NK cells expressing Ly49 receptors regulated by an NK cell-intrinsic mechanism would be increased in MHC I-deficient mice. We examined the Ly49 receptor repertoire of MHC I-deficient β2m KO mice and observed an increase in the proportion of NK cells expressing Ly49 receptors that are regulated in a cell-intrinsic manner (Ly49A, Ly49G2, and Ly49I). In contrast, the proportion of NK cells expressing Ly49 receptors regulated in an NK cell-extrinsic manner (Ly49C, Ly49D, and Ly49H) were the same or reduced in β2m KO mice (Fig 3D). These findings suggest that the lack of inhibitory ligands (MHC I), presumably leading to more NK cell activation, results in an increased chance of NK cells expressing cell-intrinsic Ly49 receptors.

### Distinct Signaling Pathways Upstream of SLP-76 Differentially Contribute to Ly49 Receptor Acquisition by NK Cells

**Fig 4:**
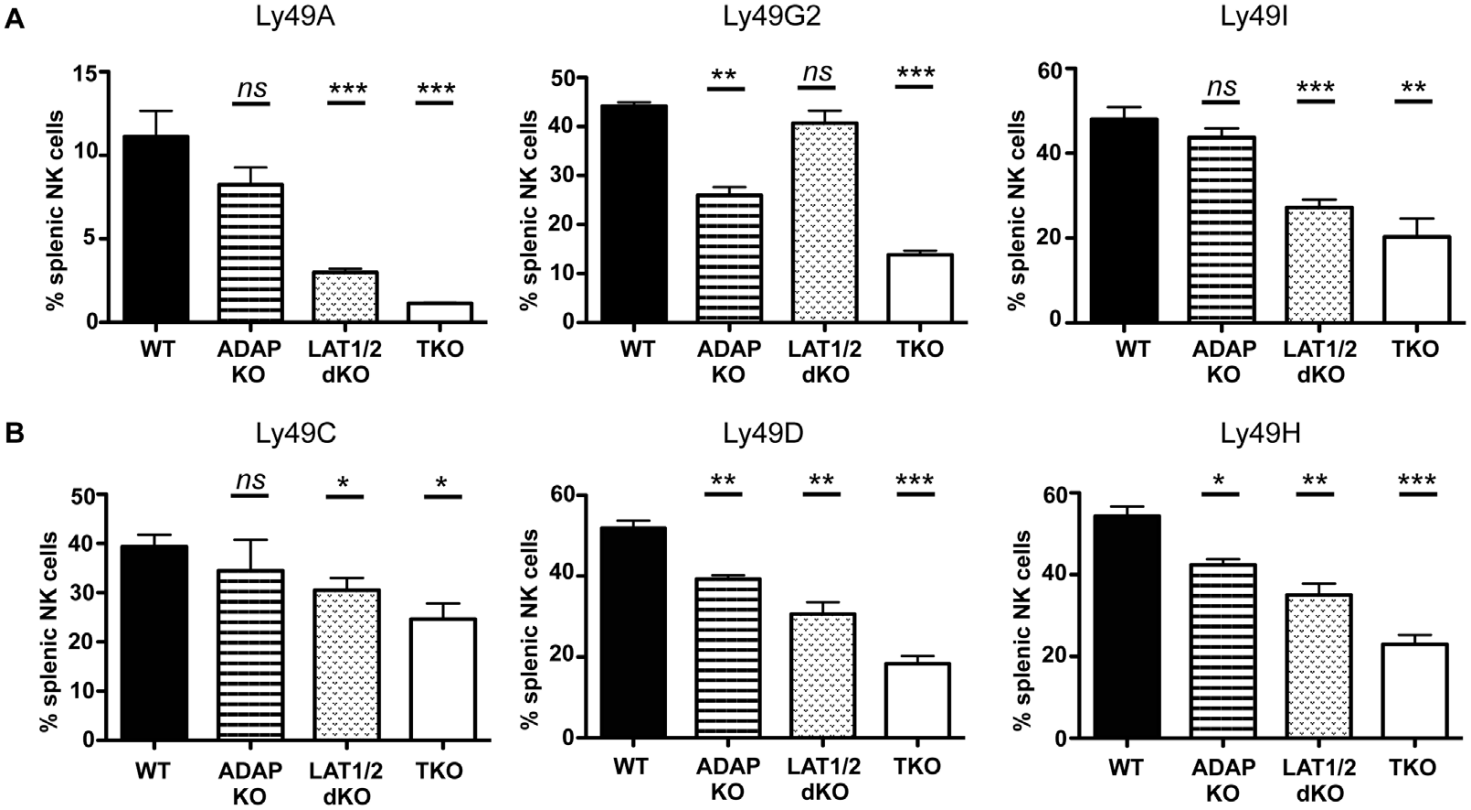
Upstream signaling pathways of SLP-76 differentially contribute to induction of Ly49 expression by NK cells. **(A)** Splenic NK cells from WT, ADAP KO, LAT1/LAT2 DKO, and ADAP/LAT1/LAT2 TKO NK cells were assessed for expression of NK cell-intrinsic Ly49A, Ly49G2, Ly49I and **(B)** NK cell-extrinsic Ly49C, Ly49D, and Ly49H. Data are represented as mean percent positive ± SEM of *n* = 3–4 mice per genotype. *p < 0.05, **p < 0.01, ***p < 0.001, and ns = not significant by student’s *t* test. See S1 Data for raw data.

In NK cells, SLP-76 can be recruited to the membrane by two independent proximal signaling complexes: one involving LAT family members (LAT1/LAT2) and the other involving ADAP [17,24]. As both SLP-76 signaling complexes are important for NK cell function downstream of activating receptors, we predicted that both LAT1/LAT2 and ADAP proteins would contribute to Ly49 receptor acquisition by NK cells. Surprisingly, we found that these SLP-76 signaling complexes differentially contributed to Ly49 receptor acquisition. A significantly decreased proportion of NK cells expressing Ly49A and Ly49I was observed in LAT1/LAT2 DKO but not in ADAP KO mice. Conversely, a significantly smaller proportion of NK cells expressing Ly49G2 was observed in ADAP KO but not LAT1/LAT2 DKO mice **(**Fig 4A**)**. The LAT1/LAT2/ADAP TKO mice displayed decreased proportions of all three Ly49 receptors similar to the SLP-76 KO NK cells. The upstream ADAP and LAT pathways also differently affected NK cell-extrinsic Ly49 receptors. Ly49C was primarily driven by LAT1/LAT2-dependent signals while Ly49D and Ly49H utilized both pathways for their expression (Fig 4B). These data point to a differential influence of SLP-76 upstream signaling pathways on Ly49 receptor induction during NK cell development.

### SLP-76 Regulates the Probabilistic Switch Function of the Ly49G Pro1 Promoter

**Fig 5:**
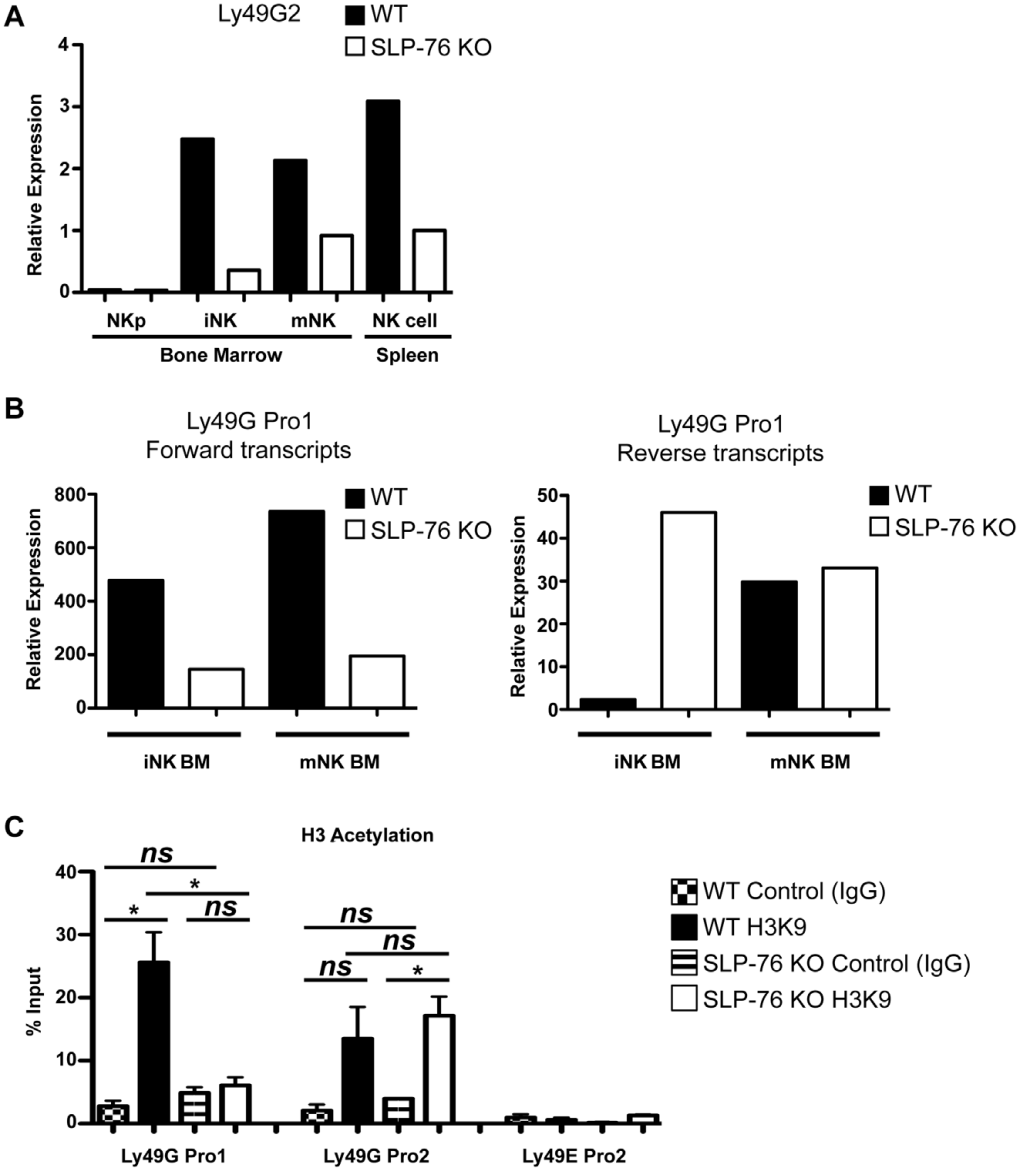
SLP-76 regulates *Ly49* gene transcription during early BM development. **(A)** qPCR was performed for Ly49G2 and GAPDH (housekeeping gene) with RNA from sorted BM NK cells at various developmental stages [CD3ε^−^CD122^+^NK1.1^−^ (NKp), CD3ε^−^CD122^+^NK1.1^+^DX5^−^ (iNK), CD3ε^−^NK1.1^+^DX5^+^ (mNK)] and splenic CD3ε^−^NK1.1^+^DX5^+^ (NK). One representative of two independent experiments is shown. (**B)** qPCR for Ly49G Pro1 forward and reverse transcripts was performed on DX5^-^ (iNK) and DX5^+^ (mNK) BM NK cells from SLP-76 KO and littermate control mice. β-actin was used as a housekeeping gene. One representative of four independent experiments is shown. (**C)** ChIP for histone 3 lysine 9 (H3K9) acetylation was performed on splenic NK cells from WT and SLP-76 KO mice. The results were normalized as the percentage of the input (%input) from Ct values, and data are represented as mean % input ± SEM from three independent experiments. *p < 0.05, **p < 0.01, ***p < 0.001, and ns = not significant by paired student’s *t* test. See S1 Data for raw data.

To test whether the reduced frequency of Ly49 receptor-expressing NK cells was due to decreased transcription, we quantified mRNA transcripts of an NK cell-intrinsic Ly49 receptor at early and late stages of NK cell development in WT and SLP-76 KO mice. We found that Ly49G2 mRNA transcripts were reduced in SLP-76 KO immature (iNK) and mature (mNK) BM subsets compared to WT controls (Fig 5A). Ly49G was chosen as a model gene since this receptor is expressed on half of NK cells.

Ly49 receptor gene transcription is driven off the Pro1 promoter region in immature NK cells and the Pro2 region during maturity [6–9]. Transcription factors can bind to Pro1 on either the positive (forward) or negative strand (reverse) in a probabilistic manner, and this determines the directionality of transcription from this promoter. Forward transcription allows for stable expression of that Ly49 receptor in mature NK cells while reverse transcription leads to no Ly49 expression. To test whether SLP-76-mediated signaling affected transcription from the Pro1 promoter, forward and reverse transcripts from the Ly49G Pro1 promoter were examined. As expected, compared to WT NK cells, DX5^−^ (BM iNK cells) and DX5^+^ (BM mNK cells) SLP-76 KO NK cells expressed significantly reduced levels of Ly49G Pro1 forward transcripts (Fig 5B). However, we also surprisingly observed that SLP-76 KO NK cells expressed increased Ly49G Pro1 reverse transcripts in iNK and mNK BM subsets compared to WT NK cells (Fig 5B). These data suggest that SLP-76-mediated signaling affects Ly49 receptor acquisition in developing NK cells by promoting Pro1 forward over reverse transcription, thereby increasing the probability of NK cells to express a given Ly49 receptor.

We further investigated the accessibility of chromatin at Ly49G Pro1 and Pro2 in WT and SLP-76 KO NK cells, by performing a chromatin immunoprecipitation for H3K9 acetylation (H3K9Ac), an indicative marker for open/accessible chromatin. The Pro2 loci of Ly49G is CpG poor and has been previously shown to be epigenetically regulated by H3K9Ac [25]. Based on the reduction in Ly49G2 expression in SLP-76 KO NK cells, we predicted there to be less H3K9Ac and less Pro2-mediated transcription. H3K9Ac was observed at Pro2 of Ly49g but not at Ly49e (silenced in adult NK cells) in WT NK cells. Surprisingly, however, Ly49G Pro2 H3K9Ac was similar between WT and SLP-76 KO NK cells (Fig 5C), perhaps suggesting that Pro2 chromatin accessibility is not sufficient to drive Ly49G expression in mature NK cells. Although the role of H3K9Ac at Pro1 is unknown, we found SLP-76 KO NK cells showed significantly decreased Ly49G Pro1 H3K9Ac compared to WT controls (Fig 5C). These data suggest that SLP-76-derived signals mainly control transcriptional activity at the Pro1 promoter and that epigenetic alterations at the Ly49 Pro1 loci may control receptor expression in mature splenic NK cells.

## Discussion

**Fig 6:**
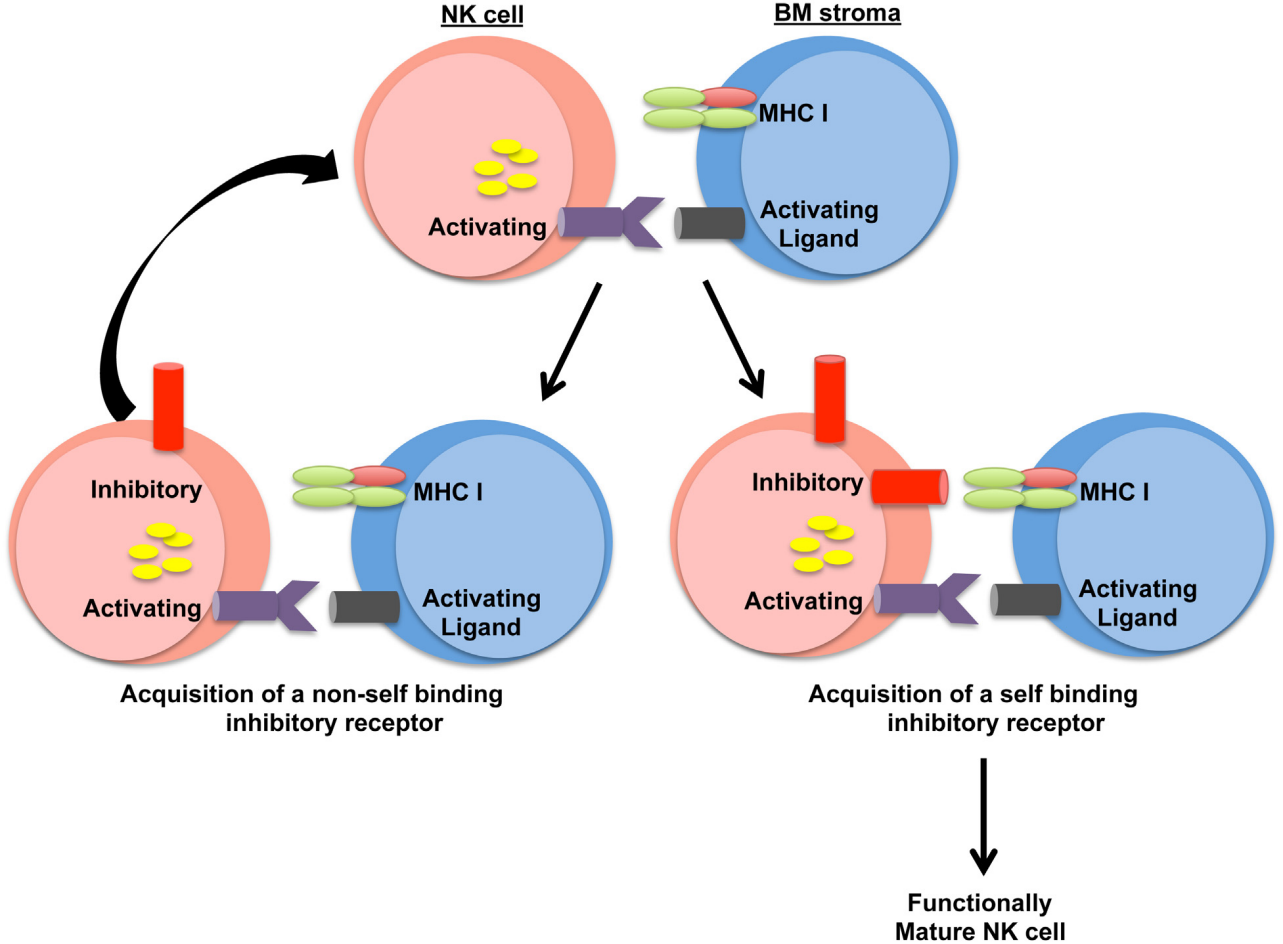
Activating receptor-mediated signaling is the driving force for Ly49 receptor induction during NK cell development. Activating receptors on developing NK cells are stimulated by their respective ligands via interactions with BM stromal cells. This interaction results in the activation of transcription factors that promote Ly49 receptor expression. If the Ly49 receptor that is acquired does not recognize self-MHC I, the NK cell continues to receive “positive” activating receptor signals, which increases the probability of the NK cell to acquire another Ly49 receptor. This process occurs until a self-MHC I binding Ly49 is acquired, which will dampen the activating receptor signal. This NK cell can then mature and migrate to peripheral tissues to perform “missing-self” recognition.

Although NK cells are part of the innate immune system, NK cells exhibit many features of adaptive immune cells. Unlike T cells and B cells that create antigen specificity by genetic recombination, NK cells create diversity by expressing a seemingly “random” assortment of inhibitory and activating receptors. The various combinations of expressed receptors generate ligand-specificity, allowing subsets of NK cells to respond, expand, and differentiate into memory-like cells in a ligand-specific manner, as well as create a diverse repertoire within the NK cell pool [26,27]. However, how NK cells determine which inhibitory receptors to express on their cell surface during a narrow window of development was largely unknown. The data presented in this manuscript support a model by which NK cell activation during development drives inhibitory receptor acquisition on immature NK cells. Our model proposes that during early NK cell development, NK cells are activated via interactions between activating receptors and their ligands expressed by BM stroma. Activation of NK cells results in a signaling cascade that promotes the transcription of different Ly49/KIR genes. The NK cell acquires these receptors until a self-binding inhibitory receptor is expressed on the cell surface and blocks the activating signal (Fig 6).

Our model potentially explains how MHC I interactions with NK cell inhibitory receptors shape the inhibitory receptor repertoire. The regulation of Ly49/KIR induction by activating receptor-derived signals provides a mechanism whereby developing NK cells can generate a ligand-specific receptor repertoire that appropriately recognizes missing self. Furthermore, the expression of a self-binding inhibitory receptor increases the functional capacity of NK cells, a process known as licensing. Thus, our model suggests that strong activating signals during NK cell development increase the likelihood of developing more functionally active licensed NK cells that can carry out missing self-recognition.

SLP-76 KO mice harbored an increased fraction of the most mature subset of NK cells. However, the decreased proportion of Ly49 receptor-expressing NK cells in SLP-76 KO mice could not be explained by differences in maturation, since the proportion of NK cells expressing Ly49 receptors was significantly reduced at each stage of NK cell maturation in SLP-76 KO mice. Our analysis showed that the fraction of Ly49 receptor-expressing NK cells was similar among all maturation stages except for Ly49I, which was overrepresented in the most mature NK cell subset in WT mice. Interestingly, the proportion of Ly49I-expressing NK cells was relatively preserved compared to Ly49A or Ly49G2-expressing NK cells. This could be potentially explained by the increased maturation status of SLP-76 KO NK cells, since almost all Ly49I-positive NK cells in SLP-76 KO mice resided in the most mature NK cell subset. Alternatively, self-binding Ly49 receptors such as Ly49I might drive NK cell maturation, yielding more mature NK cells in SLP-76 KO mice. Further investigation is required to understand how SLP-76-derived signals and self-MHC I binding Ly49 receptors impact NK cell maturation.

We were surprised to find that not all Ly49 receptor acquisition was intrinsically driven by SLP-76 signals in NK cells. While Ly49A, Ly49G2, and Ly49I were acquired in a SLP-76-dependent NK-cell intrinsic manner, Ly49C, Ly49D, and Ly49H were regulated in an NK cell-extrinsic manner. This suggests another cell type is necessary to generate a full Ly49 receptor repertoire. It is possible that myeloid lineage cells such as dendritic cells (DCs) may be responsible for NK cell activation that leads to Ly49 receptor expression [28]. NK cells and DCs form stimulatory synapses, resulting in IL-12 secretion and IL-15 transpresentation. IL-12 is critical for optimal NK cell activation by DCs, and IL-15 is required for NK cell survival and Ly49 receptor expression [29]. Since SLP-76 is critical for murine DC migration and cell–cell contact [30], the interaction of DCs with NK cells may be important for cell-extrinsic Ly49 receptor expression.

We previously reported that LAT1/LAT2 and ADAP can independently recruit SLP-76 to NK cell activation synapses [17]. As both signaling pathways are required for optimal NK cell activation, we predicted that they would equally contribute to Ly49 receptor acquisition by developing NK cells. However, LAT1/LAT2 was more important for Ly49A and Ly49I, whereas ADAP contributed to Ly49G2 expression. LAT1/LAT2 signaling pathways primarily contributed to the extrinsically regulated Ly49C and partially impacted Ly49D and Ly49H. ADAP only contributed to Ly49D and Ly49H. The Ly49 receptor phenotype of ADAP KO NK cells is supported from recently published data on SLP-76^ace/ace^ mice that contain a mutation in the SH2 domain of SLP-76, where ADAP binds [31]. Perhaps, activating receptors that preferentially use either ADAP or LAT1/LAT2 to recruit SLP-76 are engaged at different times during NK cell development, leading to the expression of Ly49 receptors in a specific order. Alternatively, each Ly49 receptor Pro1 promoter may be differentially affected by the assortment of transcription factors induced by the ADAP or LAT1/LAT2 signaling pathways.

Alteration of the probabilistic switch function of the Ly49 Pro1 promoter provides a mechanism that explains how SLP-76 signaling could increase Ly49 receptor acquisition. Ly49 receptor expression has been shown to occur in a stochastic manner [32,33], and the probabilistic mechanism has been explained by differential binding of transcription factors to either forward or reverse promoter elements in the Pro1 bidirectional promoter. Transcription factors, such as NFκB, bind this region and are inducible following activating receptor signaling. One study has explored the role of NFκB in NK cell development and Ly49 receptor expression, but the results showed only a small decrease in the proportion of Ly49 receptor-expressing NK cells [34]. Other transcription factors, such as AML and Ets-1, are expressed in NK cell progenitors prior to Ly49 receptor transcriptional initiation, but it is possible that activation induced signals and other transcription factors contribute to the stabilization of the transcriptional landscape. For example, calcium-dependent NFAT and CREB binding sites in the Pro1 region of Ly49G2 may contribute to forward transcription. Studies examining the contribution of calcium signaling to Ly49 receptor acquisition by NK cells are currently ongoing.

Since Pro1 forward transcription is thought to control the accessibility of Pro2 in mature NK cells, we predicted that SLP-76 KO NK cells would exhibit decreased Pro2 chromatin accessibility as measured by H3K9Ac at Ly49G Pro2. However, we found that SLP-76 KO and WT NK cells exhibited equivalent H3K9Ac at Pro2, despite decreased Ly49G transcripts in SLP-76 KO NK cells. Instead, we found that H3K9Ac at Pro1 was almost absent in SLP-76 KO NK cells, suggesting that SLP-76-derived signals impacted Pro1 accessibility. This suggests that Pro1 accessibility might be important for Ly49 receptor transcription in mature NK cells and that Pro2 accessibility alone is insufficient to drive Ly49 transcription. This is in line with a recent report proposing that in addition to being a bidirectional switch, Pro1 may act as an enhancer for Ly49 receptor expression in mature NK cells [35]. Further studies will be required to elucidate the exact role of Pro1 H3K9Ac in control of Ly49 receptor expression.

SLP-76-silenced human NK cells differentiated in culture were defective in human killer-cell immunoglobulin-like receptor (KIR) acquisition. Although the in vitro differentiation system may not precisely recapitulate human NK cell development in vivo, these data suggest that KIR acquisition might be influenced by SLP-76-dependent signals. It has recently been shown that KIR expression may be transcriptionally regulated in a manner similar to Ly49 receptors. There are promoter regions in KIRs similar to the Pro1 and Pro2 elements of *Ly49* genes; however, their relative location is inverted. A unidirectional promoter/enhancer is located upstream, and a proximal Pro1-like region near the transcriptional start site is methylated in nonexpressed KIRs [36,37]. The bidirectional proximal promoter has putative binding sites for transcription factors such as Sp1 and YY1 [38]. It is thought that the antisense transcripts generated from the proximal switch produce a small RNA that is involved in the transcriptional silencing of KIRs through methylation of the proximal promoter region [39,40]. The presence of a bidirectional switch in human KIRs and murine Ly49s suggests a conserved regulatory mechanism of inhibitory receptor acquisition by NK cells. Thus, our studies on activation signals driving Ly49 and KIR expression may also shed light on mechanisms by which KIRs are acquired on human NK cells.

Further investigation is needed to determine the exact transcription factors required for inhibitory receptor acquisition, as differences in the proximal signaling pathways suggest differential regulation of the Ly49 receptors. Nevertheless, our work highlights the complexity of Ly49 regulation. The results from this study are likely to be applicable to the regulation of human KIR receptors [41–43]. Our data support a model where competing activating and inhibitory receptor signals determine the probability of inhibitory receptor expression, which ultimately shapes the inhibitory receptor repertoire during NK cell development and creates appropriate ligand-specific diversity within the NK cell pool.

## Materials and Methods

### Ethics Statement

Human NK cell studies: The IRB is the University of Minnesota Institutional Review Board; Study Number: 9709M00134; Principal Investigator: Jeffrey Miller. For mouse studies, mice were euthanized using carbon dioxide according to our IACUC protocol at the University of Pennsylvania (IACUC protocol#: 804703, 804245); Principal investigator: Taku Kambayashi.

### Mice

Mice were housed in pathogen-free conditions and treated in strict compliance with the Institutional Animal Care and Use Committee regulations at the University of Pennsylvania. C57BL/6 (CD45.2^+^), B6.SJL (CD45.1^+^), and β2m KO mice were purchased from The Jackson Laboratory or Charles River Laboratories. LAT1/2 DKO, ADAP KO, and SLP-76 KO mice have been previously described and were bred in our facility [44–46]. SLP-76 KO mice have been ~3 times backcrossed to C57BL/6 mice due to embryonic lethality of fully backcrossed mice and thus, littermate controls were used for all experiments. LAT1/LAT2/ADAP TKO mice were generated by crossing LAT1/2 DKO mice to ADAP KO mice. SLP-76.B10D2 KO mice were generated by crossing B10.D2 mice to SLP-76 KO mice and screening for H-2^d^ alleles. All mice were sacrificed and analyzed between 10–12 wk of age.

### Reagents and Abs

All reagents were purchased from Sigma-Aldrich (St. Louis, MO) unless otherwise specified. Cytokines were purchased from Peprotech (Rocky Hill, NJ). Abs for cell stimulation were purchased from BioXcell (Malaysia) or Biolegend. Abs for flow cytometry were purchased from Biolegend, eBiosciences, BD Biosciences, and Molecular Probes. The following Ly49 receptor antibodies/clones were used: Anti-Ly49A (YE1/48.10.6), anti-Ly49G2 (4D11), anti-Ly49I (YL1-90), anti-Ly49D (4E5), anti-Ly49H (3D10), and anti-Ly49C/I (5E6) from BD Pharmigen. Anti-Ly49C (4LO3311) was purchased from the UCSF Cell Culture Facility (San Francisco, CA).

### Flow Cytometry, Cell Sorting, and Data Analysis

Cells were stained with antibodies against cell-surface antigens and LIVE/DEAD cell stain at 4°C for 20 min. Intracellular staining was performed using the Cytofix/Cytoperm Fixation and Permabilization Kit (BD Pharmingen) per manufacturer instructions. Flow cytometry was performed with a FACS Canto flow cytometer (BD Biosciences), and cell sorting was performed using a FACSAria (BD Biosciences). Data were analyzed with FlowJo software (TreeStar, Ashland, OR) and Simplified Presentation of Incredibly Complex Evaluations (SPICE- NIAID, Bethesda, MD). Statistical analysis was performed using Prism (GraphPad, San Diego, CA) computer software.

### Primary NK Cell Cultures and Stimulations

Splenocytes were plated in 96-well plates in NK-cell media (MEMα[Invitrogen] with 10% FBS, 1% penicillin/streptomycin, 10 mM HEPES and 1 x 10^−5^ β-ME) with human IL-2 (1,000 U/mL) on plate-immobilized (20 ug/mL) anti-NK1.1, anti-NKG2D, anti-Ly49H, anti-CD244 or with soluble PMA (100 ng/mL) and ionomycin (1 ug/mL) in the presence of monensin (eBiosciences) and anti-CD107a-PE for 6 h at 37°C. Following incubation, IFNγ production and degranulation were analyzed by flow cytometry.

### Generation of Mixed BM Chimeras

SLP-76 KO (CD45.1^+^) BM cells were T/NK cell depleted by CD3 and NK1.1 magnetic bead depletion (Miltenyi Biotec). T/NK-cell depleted BM cells from CD45.1^+^CD45.2^+^ WT (competitor) mice were mixed at a 2:1 ratio with the SLP-76 KO BM. Cells were injected intravenously into lethally irradiated (950 cGy) CD45.2^+^ recipient mice. Mixed BM chimeric mice were analyzed by flow cytometry 10–12 wk post injection.

### RT-PCR

RNA was purified from equivalent cell numbers of sorted splenic and BM NK cells (RNeasy kit-Qiagen). cDNA synthesis was performed using SuperScript II Reverse Transcriptase (Invitrogen) kit and performed using manufacturer’s instructions. Primers for SLP-76, Ly49G2, and Ly49I (Applied Biosystems) were used. The reaction was performed on the Applied Biosystems StepOnePlus Real Time PCR System (Carlsbad, CA), and ΔΔ-CT method was employed. Results were normalized to the housekeeping gene GAPDH.

### RT-PCR of Ly49G2 Pro1 Transcripts

Total RNA was purified from 100,000 sorted cells with the RNeasy Micro Kit (Qiagen), and cDNA synthesis was carried out using Random Hexamer primer, Taqman Reverse Transcription Reagents kit (Applied Biosystems) according to the manufacturer’s instructions. The primers used in the Ly49-specific qRT-PCR assay were: Ly49g-Pro1 forward primer (5′-CAAGTGATCAGCCTATTCTTGTG-3′); Ly49g-Pro1 reverse primer (5′-CTTGTGTGAGTTTTGTACTTCAG -3′); Ly49g-Pro1as forward primer (5′-CACTGCCTTATATGCCTAAACAC-3′); Ly49g-Pro1as reverse primer (5′-GACTTCATGACTAGTTACTGG-3′); β-Actin forward primer (5′-CCTGGCACCCAGCACAAT-3′); and β-Actin reverse primer (5′-GGGCCGGACTCGTCATACT-3′). Reactions were carried out using the FastStart SYBR Green Master kit (Roche Diagnostics, Indianapolis, IN, US) on the 7300 Real-Time PCR System (Applied Biosystems). The qRT-PCR was performed in duplicate and was repeated in at least three separate experiments using the following conditions. Reaction mixtures contained 12.5 μL of SYBR Green master mix, 2 pmoles each of forward and reverse primers and 5 ng cDNA. Thermocycler conditions included initial denaturation at 50 and 95°C (10 min each), followed by 40 cycles at 95°C (15 s) and 60°C (1 min). Melting curve analyses were performed to verify the amplification specificity. Relative quantification of gene expression was performed according to the ΔΔ-CT method using the StepOne Software 2.0 (Applied Biosystems). The results were normalized to the housekeeping gene β-Actin.

### Chromatin Immunoprecipitation (ChIP)

WT and SLP-76 KO splenic NK cells (CD3ε ^−^NK1.1^+^NKp46^+^DX5^+^) were sorted by flow cytometry, cross-linked for 10 min with 1% formaldehyde in cold PBS buffer, and subsequently quenched with 125 mM glycine for 5 min. The cells were pelleted by centrifugation at 470× g for 10 min and washed with PBS containing protease inhibitor cocktail. After centrifugation, the supernatant was discarded, and the cell pellet was stored at −80°C. DNA shearing was performed with a chromatin immunoprecipitation enzymatic shearing kit (Chromatrap, Ashland, VA) following the manufacturer’s instructions. Immunoprecipitation was performed with the True MicroChIP kit (Diagenode, Denville, NJ, USA) using a CHIP grade antibody against H3K9ac (Diagenode). A nonspecific rabbit IgG was used as a negative control. All ChIP steps were performed in Eppendorf 1.5-ml DNA LoBind Tubes (Eppendorf, Hamburg, Germany). The specific primer sequences used in ChIP-qPCR were as follows: Ly49g Pro-1, forward, 5’- CCCATCAAGGACTATGTGTTTAGG-3’, reverse,5’-ATGGTAAACTTCACAGATCTTAGG-3’; Ly49g Pro-2, forward, 5’-CACAGGAATCACTTCTCAGTAGA-3’,reverse, 5’-ATCGAGCGCTCACATAACACTAT-3’;Ly49e Pro-2, forward, 5’-GCAATTTCCTCCTTTTGCTTAGATA-3’,reverse, 5’-TGGAGGGAAAAGTTGGGTGAAA-3’. The precipitated DNA fractions were quantified by real-time PCR with the FastStart Universal SYBR Green Master Kit (Roche Diagnostics, Indianapolis, IN, USA) using 7300 Real-Time PCR System; (Applied Biosystems). The results were normalized as the percentage of the input (%input) from Ct values. The experiments were repeated three times.

### CD34^+^ Cell Retroviral Transduction and In Vitro Development

The use of all human tissue was approved by the Committee on the Use of Human Subjects in research at that University of Minnesota (Minneapolis, MN), and informed consent was obtained in accordance with the Declaration of Helsinki. Lentivirus containing either scramble control or SLP-76 shRNA in pGIPZ vectors was packaged in 293T cells using PAX2 and pMDG.2 plasmids (Open Biosystems, Lafayette, CO). A pool of four SLP-76 shRNA vectors (clones V2LHS_62885, V3LHS_364697, V2LHS_62886 and V3LHS_364699) was used. CD34^+^ hematopoietic cells were isolated from umbilical cord blood by double-column positive selection using anti-CD34 microbeads (Miltenyi Biotec). Cells were transduced with lentivirus by spin transduction, and CD34^+^GFP^+^ cells were sorted. CD34^+^GFP^+^ cells were cultured for 21 days on the EL08-1D2 fetal stromal line [47]. The culture media and cytokines used for human NK cell differentiation are published [48].

## Supporting Information

S1 Data Individual values used for quantification in the text, figures, and supplementary materials. (XLSX)

S1 Fig Ly49 receptor expression is reduced at all stages of NK cell splenic maturation. Ly49 receptor expression was assessed at three stages of splenic NK cell maturation in WT (black bars) and SLP-76 KO (white bars) mice. Maturity in the spleen evolves as follows: CD27^+^CD11b^−^ (least mature) → CD27^+^CD11b^+^ → CD27^−^CD11b^+^ (most mature). Data is represented as percent positive ± SEM of three independent experiments (*n* = 3 mice). *p < 0.05, **p < 0.01, ***p < 0.001, and ns = not significant by unpaired student’s *t* test.
(TIF)

S2 Fig Human NK cells require SLP-76 for optimal KIR acquisition during development. Knockdown of SLP-76 from differentiated human NK cells transduced with scramble (black bars) or SLP-76 shRNA (white bars) at Day 21 culture is shown. SLP-76 MFI was calculated from scramble or SLP-76 shRNA transduced donors. Data is plotted as MFI ± SEM of two independent experiments (*n* = 5 donors over two experiments). *p < 0.05, ***p < 0.001, by paired student’s *t* test. (B) Representative flow plots and histograms of CD56^+^CD3^-^, NKp46^+^ and KIR^+^ (KIR2DL1, KIR2DL2/DL3, KIR3DL1 antibody cocktail) NK cells are represented as mean percent positive ± SEM of two independent experiments (*n* = 5 donors over two experiments). *p < 0.05, ***p < 0.001, by paired student’s *t* test.
(TIF)

## Abbreviations

Abs: antibodies
BM: bone marrow
CMV: cytomegalovirus
DC: dendritic cell
H3K9: histone 3 lysine 9
H3K9Ac: H3K9 acetylation
KIR: Killer immunoglobulin-like
KO: knockout
MHC I: major histocompatibility complex class I
NK: natural killer
SEM: standard error of the mean
SLP-76: SH2 domain-containing leukocyte protein-76
WT: wild-type
