# Correction: Activating Receptor Signals Drive Receptor Diversity in Developing Natural Killer Cells

**DOI:** 10.1371/journal.pbio.1002590

**Published:** 2016-12-29

**Authors:** Jacquelyn Freund, Rebecca M. May, Enjun Yang, Hongchuan Li, Matthew McCullen, Bin Zhang, Todd Lenvik, Frank Cichocki, Stephen K. Anderson, Taku Kambayashi

In Fig 2A, the color labels on the bar graph for WT and SLP-76 KO were incorrect. WT should be in red, and SLP-76 KO should be in blue. Please see the correct Fig 2 here.

**Fig 2:**
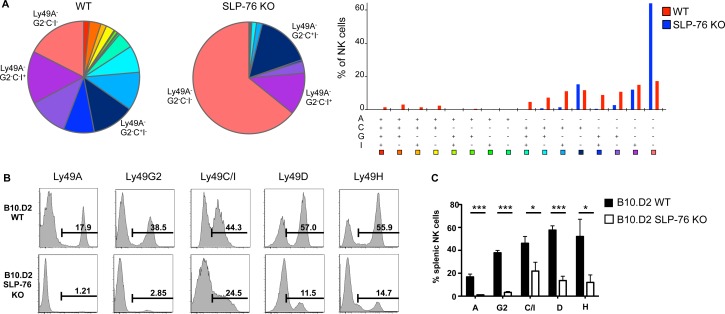
Ly49 receptor expression loss in SLP-76 KO NK cells is independent of MHC I haplotype. **(A)** The coexpression pattern of inhibitory Ly49 receptor in WT vs SLP-76 KO splenic NK cells was assessed through SPICE analysis. (**B)** Representative histograms of Ly49 receptor expression by WT B10.D2 and B10D2.SLP-76 KO splenic NK cells are shown. (**C)** The proportion of Ly49 receptor-expressing NK cells from multiple WT B10.D2 (black bars) and B10D2.SLP-76 KO (white bars) mice is represented as mean percent positive ± SEM of *n* = 3 mice/group. *p < 0.05, ***p < 0.001 by student’s *t* test. See S1 Data for raw data.
