# Supplementary figures and images for: Activating Receptor Signals Drive Receptor Diversity in Developing Natural Killer Cells
Source: PLoS Biol. 2016 Aug 8;14(8):e1002526. doi: 10.1371/journal.pbio.1002526 (PMC4976927; doi:10.1371/journal.pbio.1002526)

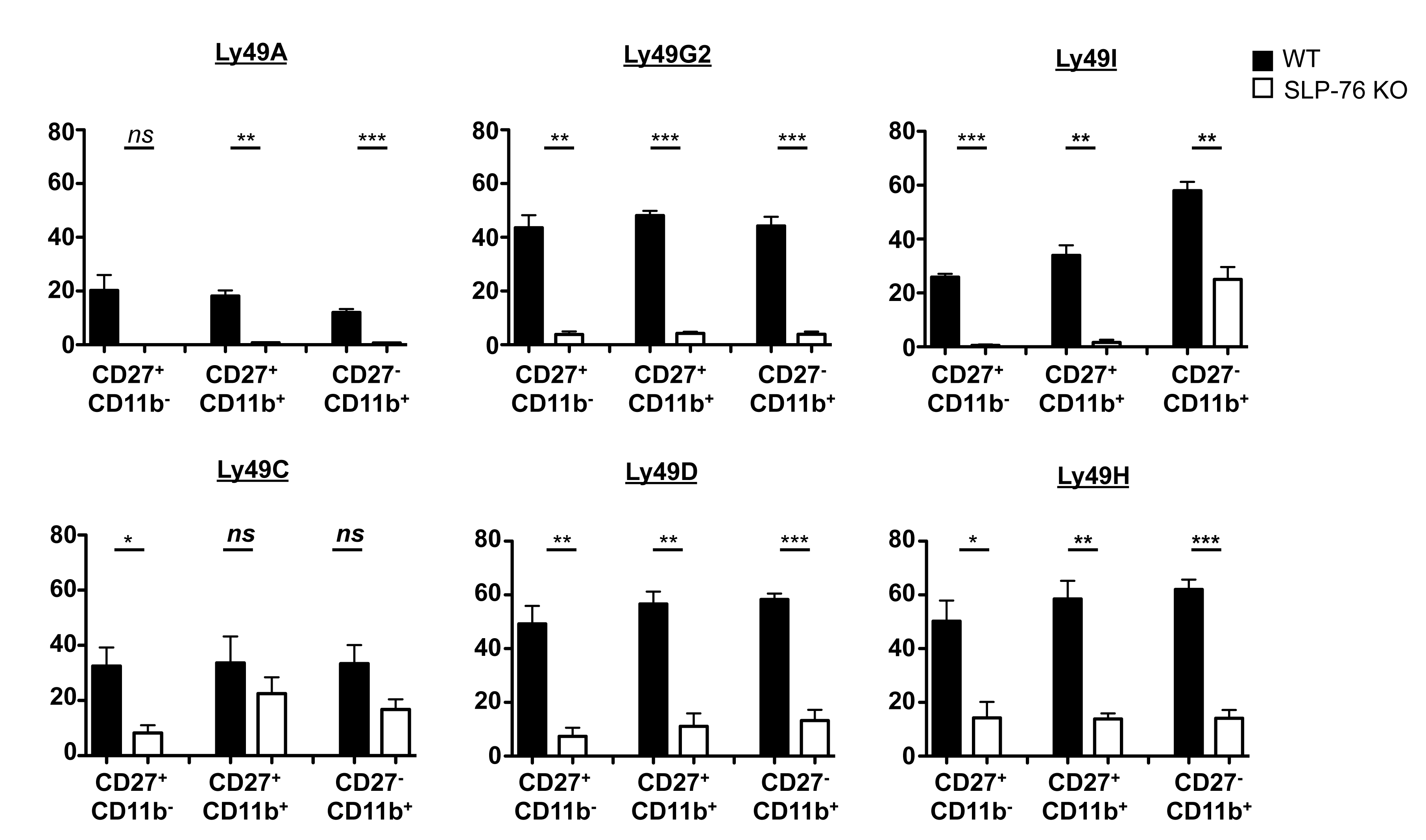

Supplement: S1 Fig — Ly49 receptor expression was assessed at three stages of splenic NK cell maturation in WT (black bars) and SLP-76 KO (white bars) mice. Maturity in the spleen evolves as follows: CD27+CD11b− (least mature) → CD27+CD11b+ → CD27−CD11b+ (most mature). Data is represented as percent positive ± SEM of three independent experiments (n = 3 mice). *p < 0.05, **p < 0.01, ***p < 0.001, and ns = not significant by unpaired student’s t test. (TIF) [file pbio.1002526.s002.tif]

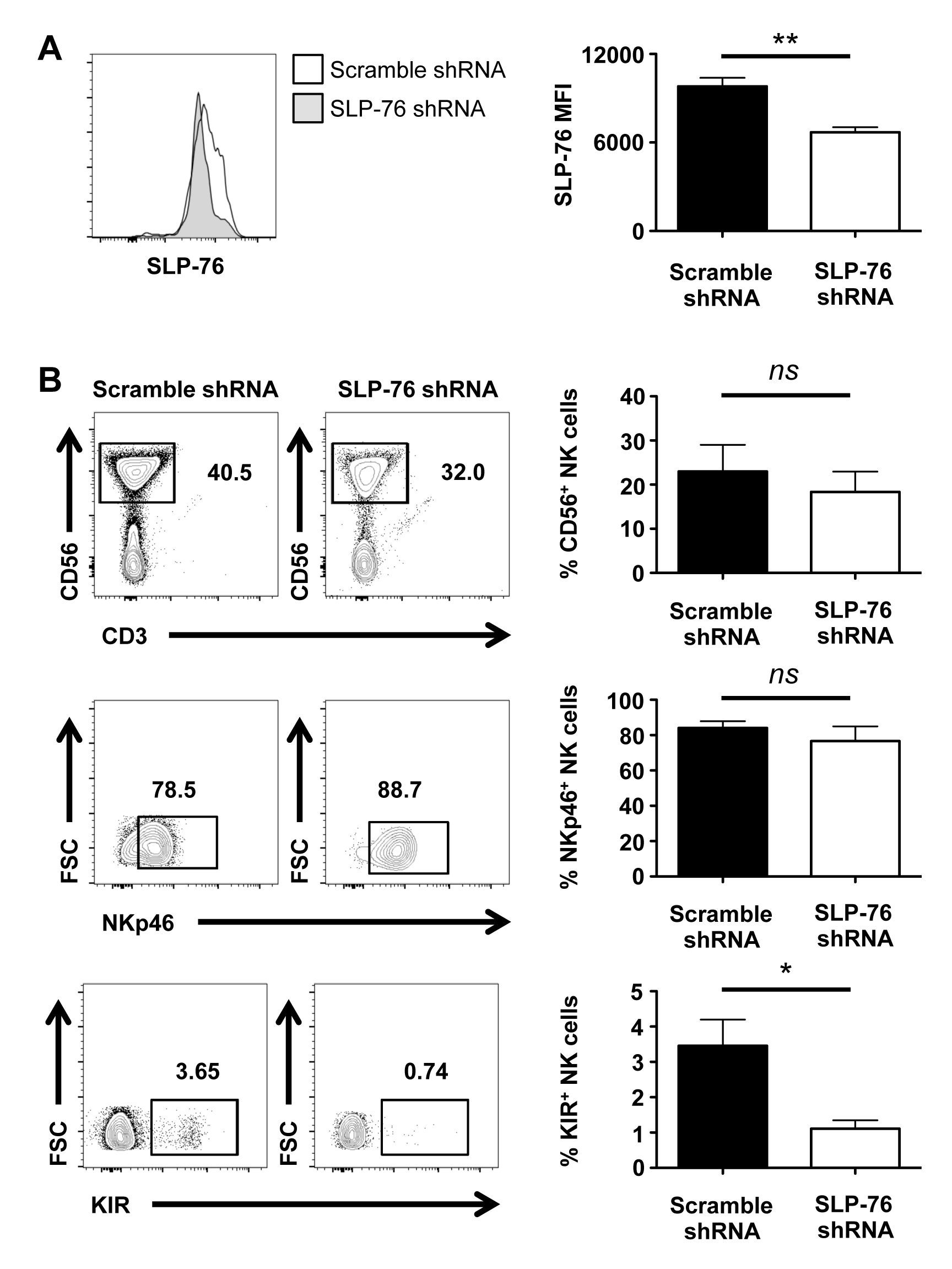

Supplement: S2 Fig — Knockdown of SLP-76 from differentiated human NK cells transduced with scramble (black bars) or SLP-76 shRNA (white bars) at Day 21 culture is shown. SLP-76 MFI was calculated from scramble or SLP-76 shRNA transduced donors. Data is plotted as MFI ± SEM of two independent experiments (n = 5 donors over two experiments). *p < 0.05, ***p < 0.001, by paired student’s t test. (B) Representative flow plots and histograms of CD56+CD3-, NKp46+ and KIR+ (KIR2DL1, KIR2DL2/DL3, KIR3DL1 antibody cocktail) NK cells are represented as mean percent positive ± SEM of two independent experiments (n = 5 donors over two experiments). *p < 0.05, ***p < 0.001, by paired student’s t test. (TIF) [file pbio.1002526.s003.tif]
